# Nonbacterial Thrombotic Endocarditis Resolved by Early Initiation of Anticoagulation Therapy and Surgical Intervention

**DOI:** 10.7759/cureus.97788

**Published:** 2025-11-25

**Authors:** Toshiki Nakamura, Satoshi Kometani, Tomohide Takei, Eri Watanabe, Miyu Sakushima

**Affiliations:** 1 Anesthesiology, Yamato Seiwa Hospital, Yamato, JPN

**Keywords:** acute arterial thrombosis, hypercoagulable state, nonbacterial thrombotic endocarditis (nbte), therapeutic anticoagulation, transesophageal echocardiography (tee)

## Abstract

Nonbacterial thrombotic endocarditis (NBTE) is a rare hypercoagulable condition, often associated with malignancy, sepsis, and autoimmune diseases, and carries a poor prognosis. This report presents a case of NBTE in the absence of an identifiable underlying disease, successfully treated with early anticoagulant therapy and surgical intervention. A 70-year-old male presented with a right upper extremity arterial occlusion and concomitant acute myocardial infarction. Transesophageal echocardiography revealed thrombus-like lesions in the left atrial appendage and aortic valve. With negative blood cultures and no clear etiology, NBTE was suspected based on systemic thromboembolic symptoms and echocardiographic findings. Anticoagulation therapy was initiated, followed by surgical removal of the left atrial appendage thrombus and coronary artery bypass grafting. Pathological examination confirmed the presence of sterile thrombi, consistent with NBTE. This case highlights the importance of considering NBTE in patients with multiple systemic emboli and negative blood cultures, even in the absence of an obvious underlying disease. It also emphasizes the benefits of prompt anticoagulant therapy and surgical intervention for improving patient outcomes.

## Introduction

Nonbacterial thrombotic endocarditis (NBTE) is a rare disease characterized by a hypercoagulable state, multiple thrombus formations, and poor prognosis. The reported prevalence of NBTE ranges from 0.3% to 9.0%, which is thought to vary depending on underlying conditions and diagnostic methods [1]. It is also associated with malignancies, sepsis, and autoimmune diseases [2]. NBTE is frequently accompanied by embolic events, with previous reports indicating that 54% of patients develop cerebral infarction within one month of diagnosis, and the one-year mortality rate is 33% [3]. However, the diagnosis is often challenging, and in many cases, the disease is first noted during autopsy. Management of NBTE generally involves treatment of the underlying condition together with anticoagulation therapy, whereas the indication for surgical intervention should be determined on an individual basis [4]. In this report, we describe a case of NBTE without any underlying complications that was successfully treated with early anticoagulation and surgical intervention.

## Case presentation

A 70-year-old male with a history of self-interruption of treatment for type 2 diabetes mellitus visited a local clinic complaining of sudden numbness, weakness, and coldness in his right arm. Ankle brachial index measurement suggested acute right upper extremity arterial occlusion, and the patient was transferred to our hospital for close examination and treatment. On presentation, the patient was alert and afebrile. Vital signs were as follows: body temperature of 36.3°C; blood pressure of 105/74 mmHg; heart rate of 98 beats/min; respiratory rate of 15 breaths/min; and oxygen saturation of 98% on room air. The patient had no recent history suggestive of viral infection and no exposure to heparin prior to admission. Laboratory findings on admission are summarized in Table 1.

**Table 1:** Laboratory findings on admission. PT-INR: prothrombin time-international normalized ratio; APTT: activated partial thromboplastin time; FDP: fibrin degradation product.

Parameter	Result	Reference range	Unit
White blood cells	8.4	3.5 - 9.0	×10⁹/L
Red blood cells	4.9	4.3 - 5.7	×10¹²/L
Hemoglobin	136	135 - 175	g/L
Platelets	229	150 - 400	×10⁹/L
PT-INR	0.98	0.8 - 1.2	-
APTT	28.3	25 - 35	seconds
FDP	19.6	<5	μg/mL
Fibrinogen	4.9	2.0 - 4.0	g/L

**Figure 1:**
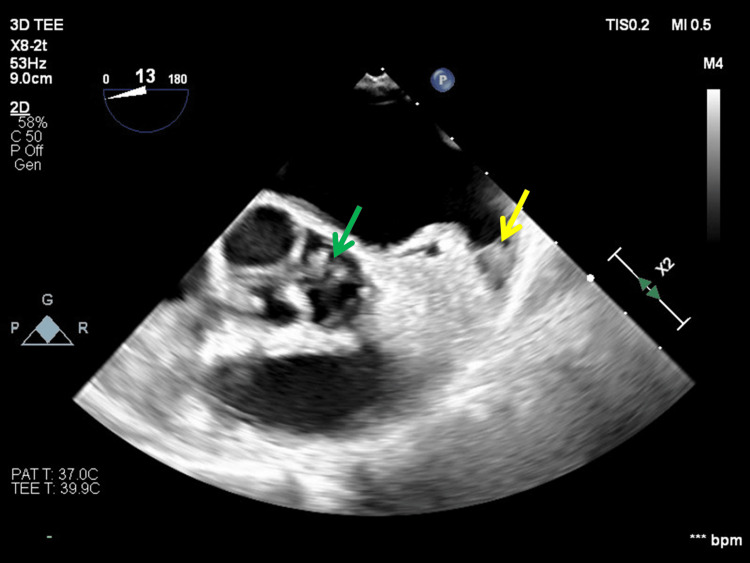
Transesophageal echocardiography showing mass lesions within the left atrial appendage (yellow arrow) and the aortic valve (green arrow).

Laboratory tests revealed activation of the fibrinolytic system, suggestive of thrombus formation with fibrin/fibrinogen degradation products at 19.6 µg/mL (reference value: <5.0 µg/mL), and the contrast-enhanced computed tomography scan revealed right brachial artery thrombosis. A diagnosis of acute brachial artery occlusion was confirmed, and emergency surgery was scheduled. However, as ST-segment elevations were observed upon arrival in leads II, III, and aVF on electrocardiography, coronary angiography was performed first, which revealed complete occlusion of the right coronary artery. Due to the higher urgency of the coronary artery lesion, percutaneous coronary intervention (PCI) was first performed by an Ultimaster Nagomi stent (2.25 mm × 38 mm, Terumo, Tokyo, Japan). Subsequently, general anesthesia was administered to remove the thrombus from the right brachial artery. The thrombus was retrieved using a 4-Fr Fogarty catheter and consisted of a mixture of white and red thrombi. Transesophageal echocardiography (TEE), performed intraoperatively to assess the degree of cardiac dysfunction resulting from the myocardial infarction, coincidentally revealed tumor-like lesions suspected to be thrombi in the left atrial appendage and left coronary cusp of the aortic valve without regurgitation (Figure 1).

Based on these systemic thromboembolic manifestations, including cardiac involvement and blood test findings, the patient was considered to be in a hypercoagulable state. However, its underlying etiology remained unknown. The absence of a history of atrial fibrillation suggested that the thrombus formation in the left atrial appendage was due to an atypical mechanism. Although infective endocarditis was included in the differential diagnosis, there was no evidence of leaflet destruction or regurgitation in any valve, and the patient did not fulfill the Duke diagnostic criteria [5]. Heparin-induced thrombocytopenia (HIT) and other anti-platelet factor 4 (PF4) antibody-mediated disorders were considered in the differential diagnosis; however, HIT was not strongly suspected because the patient had no prior heparin exposure before admission and exhibited no decrease in platelet count during the clinical course. Given the low 4T’s score (three points), HIT was effectively ruled out [6].

**Figure 2:**
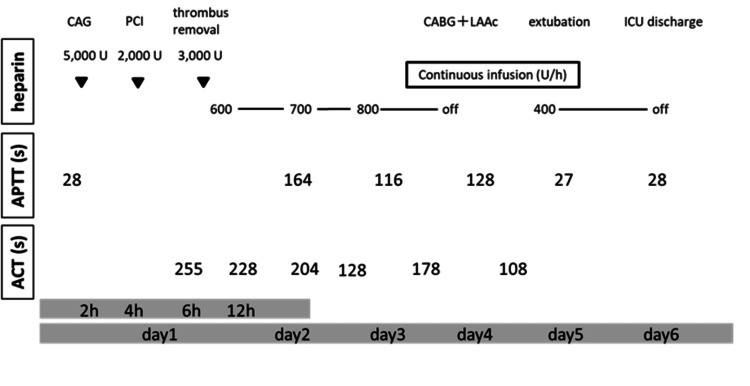
Perioperative time course from admission regarding heparin management. ACT and APTT reference ranges are 97-133 seconds and 25-40 seconds, respectively. ICU: intensive care unit; CAG: coronary angiography; PCI: percutaneous coronary intervention; CABG: coronary artery bypass grafting; LAAc: left atrial appendage closure; APTT: activated partial thromboplastin time; ACT: activated clotting time.

Therefore, NBTE could not be excluded, and treatment with unfractionated heparin was initiated with the activated clotting time maintained between 150 and 230 seconds. The perioperative time course is shown in Figure 2.

The activated clotting time (ACT) measurement device was an Actalyke Mini II (Helena Labs, Beaumont, TX), and the reagent tubes were MAX-ACT (Helena Labs).

Although the thrombus-like mass on the aortic valve leaflet gradually resolved, the thrombus persisted in the left atrial appendage. Given the patient's prior embolic events involving the coronary artery and right upper extremity, the risk of further embolization was considered high. Therefore, on the third day of hospitalization, the patient underwent left atrial appendage closure and thrombus removal via median sternotomy under cardiopulmonary bypass. Coronary artery bypass grafting was also performed for residual coronary artery lesions using the left internal mammary artery to the left anterior descending artery and the great saphenous vein to the obtuse marginal branch. The postoperative course was uneventful. The patient was weaned from mechanical ventilation on postoperative day one and discharged from the ICU on postoperative day five. Anticoagulation therapy was reinitiated on postoperative day one with continuous intravenous unfractionated heparin, which was later transitioned to oral warfarin, targeting the prothrombin time-international normalized ratio (PT-INR) to 1.6-2.0 (reference value: from 0.85 to 1.15).

**Figure 3:**
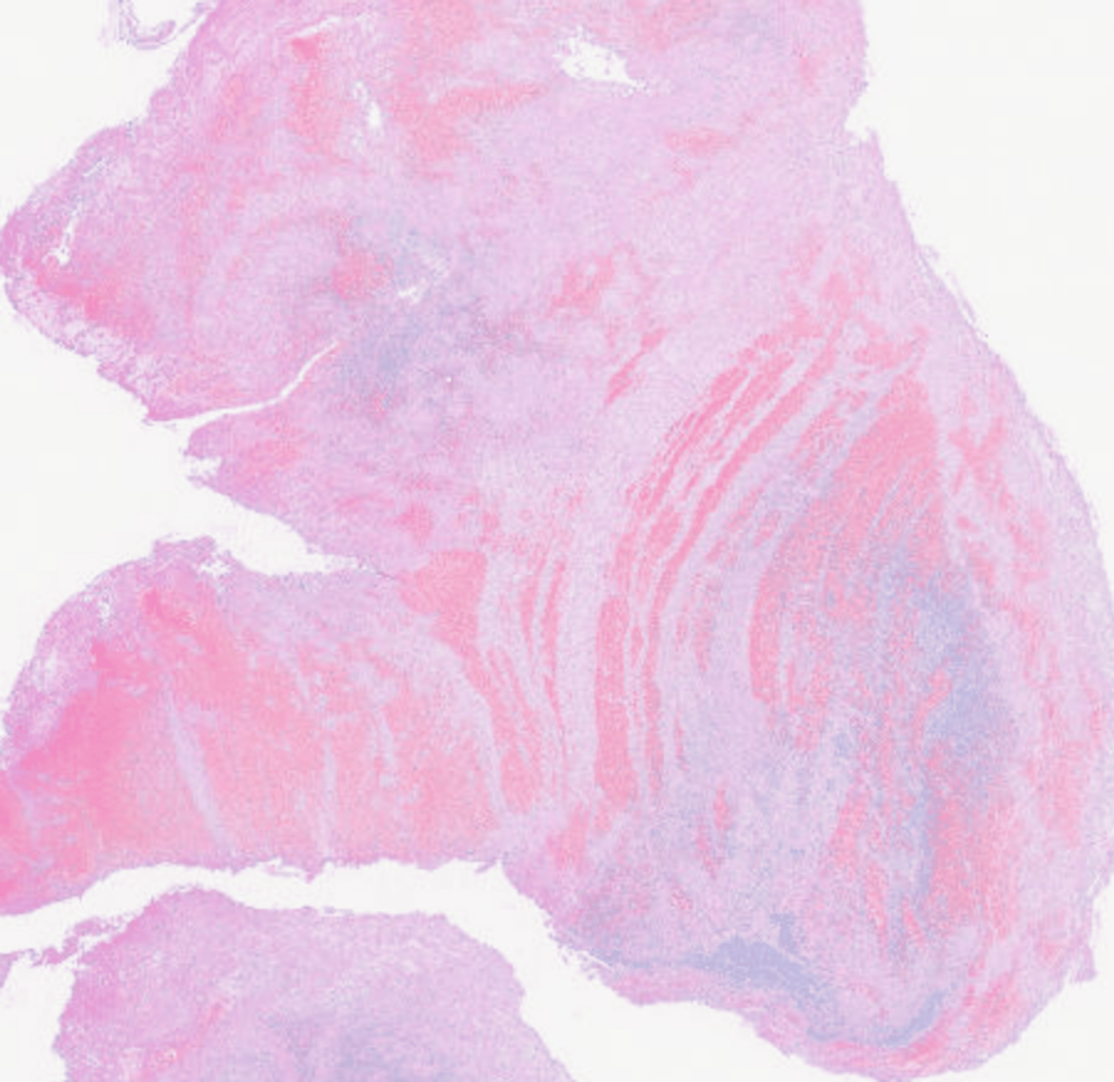
Pathological examination of the collected specimen of the left atrial appendage confirmed fresh, sterile thrombi composed of platelets and fibrin.

Warfarin therapy was continued throughout the one-month hospital stay and subsequently transitioned to a direct oral anticoagulant upon discharge. No thrombus recurrence was observed during hospitalization. All blood cultures obtained during treatment were negative. The thrombi retrieved during PCI and surgery were subjected to pathological examination. All specimens were identified as fresh, sterile thrombi composed primarily of platelets and fibrin, consistent with the diagnosis of NBTE (Figure 3).

## Discussion

NBTE was first described by Ziegler (1888) as a tumorous lesion characterized by fibrin deposition in heart valves, which he termed “thromboendocarditis.” In 1924, Libman and Sacks further characterized NBTE as a distinct form of endocarditis, unlike bacterial and rheumatic types [7]. Conditions associated with NBTE include malignant tumors, autoimmune diseases such as systemic lupus erythematosus and antiphospholipid syndrome, tuberculosis, acquired immunodeficiency syndrome (AIDS), uremia, sepsis, severe trauma, and advanced age. In-hospital mortality of NBTE has been reported to be as high as 30%. Common causes of death in patients with NBTE include cerebral infarction, myocardial infarction, and pulmonary embolism [2].

NBTE is typically diagnosed in the presence of underlying conditions, including malignancies and autoimmune diseases, when blood cultures are negative, and the vegetations identified on the valve leaflets are sterile. The sensitivity of transthoracic echocardiography (TTE) for detecting vegetation varies from 40% to 70%, whereas TEE offers a consistent sensitivity of 90% to 95% [8]. Autopsy studies have demonstrated that 75% of vegetations on the cardiac valves are less than 3 mm in size, which likely contributes to the superior diagnostic accuracy of TEE over TTE [1]. NBTE is often associated with multiple embolic events in the brain, kidneys, spleen, and extremities. Cerebral infarction, including transient ischemic attack, occurs in approximately 70% of the cases, whereas myocardial infarction occurs in approximately 15% [2]. Therefore, NBTE should be considered in cases of multiple systemic emboli with negative blood cultures and vegetations that do not respond to antimicrobial therapy. Following the diagnosis of NBTE, immediate initiation of anticoagulation therapy with heparin is advised; nevertheless, in the presence of cerebral infarction, attention must be paid to the risk of hemorrhagic transformation. The appropriate duration of anticoagulation remains undefined: while it may be discontinued after the resolution of malignancy, lifelong therapy is generally recommended in patients with advanced cancer or autoimmune disorders [4]. Although there are no definitive guidelines for surgical intervention in NBTE, surgery may be indicated in cases involving highly mobile vegetations, significant valvular dysfunction, or failure to respond to anticoagulation therapy [4,9].

In the present case, tests for collagen disease-related autoantibodies and tumor markers yielded negative results. There was no clinical history or laboratory evidence suggestive of HIT or PF4 antibodies, and platelet counts remained within the normal range. Since no other underlying disorder was identified, establishing a definitive diagnosis was challenging. However, the presence of multiple embolic events consistent with a hypercoagulable state, along with TEE findings, supported the diagnosis of NBTE. As TEE is an invasive modality, it is typically reserved for cases in which TTE fails to provide a definitive diagnosis. In the present case, the urgency of catheterization and subsequent surgery precluded a detailed TTE assessment; nevertheless, intraoperative TEE proved to be instrumental in establishing the diagnosis and guiding management. In addition, early initiation of anticoagulation therapy and timely surgical removal of the thrombus likely contribute to favorable outcomes in this life-threatening condition, which is associated with a high mortality rate.

## Conclusions

The diagnosis of NBTE is challenging. It should be considered in patients presenting with systemic emboli or a hypercoagulable state in the absence of an obvious cause, such as atrial fibrillation or infective endocarditis. The identification of intracardiac vegetations via echocardiography may influence therapeutic decision-making. Diagnostic tests such as autoimmune antibody panels and tumor markers, which may contribute to the diagnosis of NBTE, often require external laboratory processing depending on the facility, and the results may not be immediately available. In life-threatening situations, initiating anticoagulation therapy and performing immediate surgical thrombus removal are crucial.
